# Dr. M. Elizabeth Schugens

**Published:** 1908-02

**Authors:** 


### OBITUARY.

Dr. M. Elizabeth Schugens, of Buffalo, died at her home 378 Ellicott Street, Christmas morning, December 25, 1907, after a brief illness. She was the daughter of Otto Schugens, who for many years kept a music store on Genesee Street, until his death a few years ago. Dr. Schugens was a graduate of Buffalo State

Normal School, where she prepared for her life work, that of teacher. She began her career as a teacher in School No. 36, but was soon afterward transferred to that part of Central High School which later became Masten Park High School, where she remained until her last illness. Her teaching career comprised a period of about eighteen years, during which time she not only became one of Buffalo's most successful teachers, but also one of the most respected and beloved members of Masten Park High School faculty.

She sought to increase her value as a teacher by taking the degree of doctor of medicine. Accordingly, she entered the medical department of the University of Buffalo, where after four years training she graduated with the class of 1901, and took the state license to practise medicine in the same year. It was not her intent, however, to engage in the practice of the profession of medicine, but to follow the profession of teaching for which she was fitted in high degree.

She was deeply concerned in securing medical inspection in the schools and her last public speech was made at the hearing before the councilmen on the subject of medical inspection. She was an active member of the Women Teachers' Association, a past officer of the high school section, and a valued member of the Physicians' League of this city. She was also a member of The Scribblers. With her many other gifts Dr. Schugens coupled that of a forceful speaker. At the commencement banquet of the medical department of the University of Buffalo, in 1905, she responded in a graceful speech to the toast, "The Men," which attracted the attention of the entire assemblage.

Dr. Schugens is survived by her mother and her death leaves a vacancy in her home that never can be filled.

